# When a Budget-Friendly Drug Leads to a Premium Diagnosis: A Case Report of Severe Cholestasis Following Amoxicillin–Clavulanate Use

**DOI:** 10.1155/crhe/6781220

**Published:** 2025-11-24

**Authors:** Maureen Okafor, Joel Gabin Konlack Mekontso, Nitin Joglekar, Nikisha Pandya, John Trillo

**Affiliations:** NYC Health + Hospitals South Brooklyn Health, Brooklyn, USA

**Keywords:** amoxicillin–clavulanate hepatotoxicity, bisalbuminemia, drug-induced liver injury, drug safety awareness, medication review and reconciliation

## Abstract

Drug-induced liver injury (DILI) is a rare but significant complication of commonly prescribed medications. This case describes a 69-year-old female presenting with clinical and laboratory features of mixed type liver injury, bisalbuminemia, and pseudohyponatremia. An extensive evaluation for obstructive, autoimmune, and infectious hepatitis yielded inconclusive results, necessitating advanced imaging and a liver biopsy. The biopsy revealed chronic hepatitis of unspecified etiology. On further inquiry, the patient disclosed having used amoxicillin–clavulanate for sinusitis 4 weeks prior to presentation, implicating this medication as the probable cause of her symptoms. The patient's condition improved with supportive care, with normalization of liver function within 4 months. This case underscores the importance of obtaining a detailed medication history to avoid unnecessary and costly diagnostic evaluations. Physicians must remain vigilant about rare adverse effects of commonly prescribed drugs and recognize their characteristic clinical patterns. Early identification and management can prevent complications, reduce unnecessary interventions, and optimize patient outcomes.

## 1. Introduction

Drug-induced liver injury (DILI) is a leading cause of acute liver failure in western countries. In the absence of a diagnostic biomarker, the identification of DILI remains a challenge due to its rare presentation and variations in clinicopathological patterns reflected by drug biochemical profiles and dose [1, 2]. Even more unique is its idiosyncratic form, which presents unpredictably with dose-independent hepatotoxicity; amoxicillin–clavulanate and penicillinase-resistant penicillin predominate among antibiotic-associated acute liver injury [3, 4]. Amoxicillin–clavulanate is a *β*-lactam antibiotic that is widely available and cost-effective, making it a common drug of choice for mild-to-severe urinary, respiratory, and gastrointestinal tract infections [5]. Despite the well-tolerated and safe use of amoxicillin–clavulanate, it is rarely known to cause detrimental hepatotoxic adverse effects. Despite a growing number of reported cases, there remains very limited knowledge of the pathophysiological mechanisms and determinants of its clinical manifestation [6]. Few cases have described the complex clinical picture of idiosyncratic mixed-type hepatotoxicity in amoxicillin–clavulanate-induced liver injury. Here, we present the case of a healthy adult female with weakness, dizziness, hyponatremia, cholestasis with jaundice, and bisalbuminemia. In this report, we further explored the patterns of amoxicillin–clavulanate-induced hepatotoxicity and highlighted the need for early discovery in disease prevention.

## 2. Case Description

A 69-year-old female with a history of hypertension, hyperlipidemia, and diabetes mellitus presented to the emergency department after experiencing a fall at her primary care provider (PCP) office. Over the past month, she had experienced generalized weakness and dizziness. Two weeks prior, the patient noticed dark urine, clay-colored stools, and generalized pruritus. Approximately 3-4 days before the presentation, she developed jaundice in her eyes and skin. She reported decreased appetite, weight loss of 12 pounds over a few weeks, and one episode of nonbloody, nonbilious vomiting in the past week. She denied abdominal pain, chest pain, shortness of breath, recent travel, sick contact, hematuria, or changes in bowel habits.

Two weeks earlier, she had been evaluated at a different hospital for dizziness and a syncope episode at a bus stop and reported persistent light-headedness when standing for prolonged periods since that event. Orthostatic hypotension was observed at that time. Computed tomography (CT) of the head showed no acute intracranial pathology but revealed chronic sinusitis. She was treated with intravenous fluids, which improved her orthostatic hypotension and she was discharged with plans to follow up with PCP. She reported no personal or family history of malignancy and denied use of tobacco, alcohol, recreational drugs, or herbal remedies. The patient also had no significant allergies. Her chronic medication regimen included hydroxyzine 25 mg daily, enalapril 10 mg daily, hydrochlorothiazide 25 mg daily, alogliptin 25 mg daily, pravastatin 40 mg daily, and metformin 500 mg twice daily.

During her current admission, vital signs were within normal limits. Physical examination revealed scleral icterus and mild epigastric tenderness with a positive Murphy's sign. A 12-lead EKG revealed sinus rhythm without ischemic changes. Laboratory findings included significant elevations in total and direct bilirubin, liver enzymes, serum creatinine, and hyponatremia (Table 1). A CT scan of the head revealed partial opacification of the right sphenoid sinus, with no other acute intracranial pathology. Abdominal imaging, including ultrasonography and a CT tomography, revealed hepatic fatty infiltration without evidence of cholelithiasis or biliary ductal dilation.

Differential diagnoses included obstructive jaundice secondary to malignancy or strictures, autoimmune hepatitis, or intrinsic hepatic injury with possible pseudohyponatremia from acute liver disease. The Roussel Uclaf Causality Assessment Method (RUCAM) score was 8, making DILI probable. The initial management involved intravenous normal saline at 50 mL/h, oral cholestyramine 4g three times daily, and discontinuation of pravastatin, hydrochlorothiazide, and alogliptin. Given the inconclusive imaging results, magnetic resonance cholangiopancreatography (MRCP) was performed, revealing possible mild intrahepatic ductal strictures but no significant biliary or pancreatic ductal dilation, beaded appearance, intraluminal debris, or calculi. A comprehensive workup for viral (CMV, EBV, HIV, hepatitis A, B, C, D, and E), metabolic (ceruloplasmin and ferritin), autoimmune (antinuclear antibody (Ab), antimitochondrial Ab, anti-smooth muscle Ab, anti-LKM Ab, tissue transglutaminase Ab, and soluble liver antigen), genetic (alpha 1 anti-trypsin), and toxic (acetaminophen) etiologies was unremarkable; however, serum protein electrophoresis revealed bisalbuminemia.

Faced with this perplexing presentation, endoscopic retrograde cholangiopancreatography was considered but deferred because of the absence of significant biliary or pancreatic ductal dilation, suggestive of obstruction. Ultimately, a liver biopsy was performed, revealing chronic hepatitis with moderate portal inflammation, mild fibrosis, cholestasis, steatosis, and focal mild iron deposition (Batts and Ludwig Grade 3, Stage 1) of unspecified etiology. On further questioning, the patient disclosed taking amoxicillin/clavulanate for sinusitis 4 weeks before symptom onset.

Her condition and liver function improved with supportive care, including cholestyramine. She was discharged home on metformin, cholestyramine, and metoprolol (for blood pressure management). The diagnosis provided relief to the patient, who had been in her usual state of health and unaware that amoxicillin was the source of her condition. She was educated about the risks of self-medication, her potentially hepatotoxic medications were discontinued, and she was advised to maintain regular follow-ups with her primary care physician. Liver function tests normalized within 4 months postdischarge, allowing for the discontinuation of cholestyramine.

## 3. Discussion

DILI is a relatively uncommon condition, with its incidence varying globally based on drug-use patterns and patient demographics. Among the antibiotics, amoxicillin–clavulanate is the most consistently implicated agent, accounting for up to 24% of DILI cases enrolled in the US DILI Network registry [7]. The at-risk population includes older adults and individuals on medication for pre-existing comorbidities, as seen in this patient. Genetic predispositions and polypharmacy further compound this risk, emphasizing the need for heightened vigilance in these populations [8, 9].

Although the mechanism of DILI is not fully understood, immunological hypersensitivity has been implicated as a likely mechanism in amoxicillin–clavulanate hepatic dysfunction [10]. DILI may result from predictable dose-dependent toxicity, as observed with acetaminophen or idiosyncratic reactions, which are dose-independent and unpredictable [11]. Amoxicillin–clavulanate predominantly induces idiosyncratic mixed hepatotoxicity, which is characterized by features of both cholestasis and hepatocellular injury. Amoxicillin–clavulanate–DILI remains a diagnosis of exclusion, and the lack of a defining disease biomarker presents a challenge for distinguishing DILI from other liver diseases. As such, other differential diagnoses are explored first, and a delay in diagnosis may worsen the clinical outcomes. In patients with early stage idiosyncratic mixed-type hepatotoxicity, it is important to understand the clinicopathological patterns occurring with the use of amoxicillin–clavulanate, as well as the associated dosage, dose frequency, or treatment duration, which may determine this clinical manifestation [1]. The typical time to symptom onset is within 5–30 days postexposure, consistent with this case. It can also be subacute, with symptoms occurring several weeks following therapy [10].

At the onset, patients may present with fatigue, jaundice, pruritus, anorexia, or abdominal discomfort. Clinical features may mimic other inflammatory, autoimmune, and malignant conditions, leading to an extensive workup if not accurately recognized. For instance, immune-mediated DILI may present with features of Type IV hypersensitivity, such as high-grade fever, arthralgia, fatigue, skin rash, itching, or facial edema. Eosinophilia can also occur [4].

Elevated levels of liver enzymes, such as alanine aminotransferase (ALT), aspartate aminotransferase (AST), and alkaline phosphatase (ALP), are characteristic of DILI. The ALT/ALP ratio defines the pattern of DILI as hepatocellular (ratio > 5), cholestatic injury (ratio < 2), or mixed hepatocellular and cholestatic type (ratio 2–5). Cholestatic or mixed-type liver injury has been associated with older age and prolonged amoxicillin–clavulanate therapy [6]. Interestingly, our patient exhibited an early pattern of mixed-type liver injury, which progressed to cholestatic injury on presentation and for the remaining duration of the disease until resolution. In addition, significant hyponatremia with normal serum osmolality, likely pseudohyponatremia, was found [12]. Although rare, pseudohyponatremia has been observed in other liver diseases but not in DILI. Overall, a similar clinical case has not yet been reported in the literature.

The diagnostic workup for DILI often requires a combination of clinical suspicion, laboratory evaluation, imaging, and occasionally liver biopsy. Elevated liver enzymes, particularly disproportionate elevations in alkaline phosphatase and bilirubin, support a cholestatic pattern of injury, which is common with amoxicillin-clavulanate [13]. Bisalbuminemia, noted in this case, is an unusual finding that may suggest underlying beta-lactam-induced hepatotoxicity [14, 15]. Although acquired bisalbuminemia has been reported in beta-lactam antibiotic overdose and as a consequence of severe pancreatitis [16], its presence following therapy with appropriate doses of amoxicillin-clavulanate has not been reported. Furthermore, its role in DILI pathophysiology remains unclear. Imaging studies, such as MRCP and CT scans, are instrumental in excluding structural or obstructive causes, while a liver biopsy provides definitive histological evidence of hepatotoxicity. The histological findings in this case, chronic hepatitis with portal inflammation, cholestasis, and mild fibrosis, are not pathognomonic but reinforce the diagnosis of DILI in the context of the patient's unique clinical phenotype. This case further highlights the importance of early recognition, with an understanding that the clinical patterns of DILI may evolve with a delay in diagnosis.

Management of DILI depends on immediate discontinuation of the offending agent and other hepatotoxic medications. Supportive care, including cholestyramine use to alleviate pruritus, is often sufficient [17]. Specific treatments beyond supportive care are generally lacking, stressing the importance of prevention and early recognition. In this patient, the prompt initiation of supportive therapy facilitated recovery, with liver function levels normalizing within 4 months. Long-term follow-up is essential to ensure the resolution of liver injury, monitor for possible chronic sequelae of the disease, and guide the safe use of medications in the future.

The prognosis is good in patients with amoxicillin–clavulanate–DILI once the offending drug has been identified and discontinued. However, a 10% mortality risk from cholestatic liver failure exists in the event of poor clinical management or delayed diagnosis [1, 13, 18]. Previous studies have reported a typical recovery time within a few weeks of treatment, with chronic outcomes described as persistent hepatocellular damage lasting more than 3 months following drug withdrawal or cholestatic/mixed-type damage lasting more than 6 months [19]. Overall, survival was significantly better in patients with cholestatic or mixed-type liver injury than in those with hepatocellular damage [20]. In our patient, although complete clinical and biochemical resolution of the disease was observed after 4 months of treatment, it is still important to monitor for long term or unfavorable outcomes of DILI, including chronic liver injury, vanishing bile duct syndrome, or fulminant hepatic failure requiring liver transplantation, multiorgan failure, and death [4]. Moreover, medication surveillance in patients with prior DILI is crucial to prevent future recurrence.

In clinical practice, identifying DILI poses significant challenges, particularly when patients are taking multiple potentially hepatotoxic medications or fail to establish a direct link between symptoms and prior medication use. This challenge is compounded by a delayed presentation, as in this case, where the patient's symptoms manifested weeks after completing the course of amoxicillin–clavulanate. This report also highlights the diagnostic complexities of DILI in older adults with multiple comorbidities and the unique evolving clinical patterns of mixed type to cholestatic hepatotoxicity, bisalbuminemia, and pseudohyponatremia in acute liver disease. This emphasizes the importance of maintaining a high index of suspicion for drug-induced hepatotoxicity and the critical role of thorough medication reconciliation, including inquiries about over-the-counter drugs and herbal supplements. For clinicians, this case serves as a reminder to consider DILI in the differential diagnosis of unexplained acute liver dysfunction and to adopt a systematic approach for its evaluation and management.

## 4. Conclusion

DILI due to amoxicillin–clavulanate use is a serious cause of acute liver failure, particularly among older adults with multiple comorbidities. This case underscores the necessity of understanding the varying clinical patterns of this disease and the importance of a comprehensive medication history in evaluating patients with unexplained liver dysfunction. Early recognition and intervention, including immediate discontinuation of the offending agent and supportive care, are essential for preventing further complications and optimizing patient outcomes. Clinicians must be vigilant about the rare but serious adverse effects of commonly prescribed medications to enhance patient safety and improve diagnostic accuracy.

## Figures and Tables

**Table 1:** Laboratory findings before presentation and up to resolution of disease.

Lab tests	Two weeks prior	Initial encounter	Hospital day 12	Upon discharge (23 days later)	Four months postdischarge	Normal range
Leukocytes	8.7	9.9	9.7	7.2	6.7	4.5–10.9 × 10^9^/L
Hemoglobin	14.2	13.2	8.7	9.3	13.8	12–16 g/dL
Platelets	418	557	505	412	294	150–450 × 10^9^/L
INR	1.3	1.2	—	—	—	0.9–1.1
AST	177	51	86	212	17	< = 35 U/L
ALT	529	73	84	166	28	< = 35 U/L
ALP	229	278	210	168	104	35–104 U/L
Total bilirubin	5.3	16.8	18.6	10.9	0.3	< = 1.2 mg/dL
Direct bilirubin	—	> 10	> 10	—	0.1	0.0–0.3 mg/dL
Lipase	—	203	—	—	—	13–60 U/L
Sodium	129	119	135	137	138	136–145 mmol/L
BUN	18	31	13	11	15	8–23 mg/dL
Creatinine	1.19	1.3	0.6	0.5	0.9	0.5–0.9 mg/dL
Serum osmolality	—	283	—	—	—	270–295 mOsm/kg
Urinalysis	—	Bilirubin	—	—	—	—
ANA	—	1:160	Negative	—	—	< 1:80
Anti-SMA	—	1:80	—	—	—	< 1:20
AMA	—	< 1:20	Negative	—	—	< 1:20

Abbreviations: ALT: alanine aminotransferase; ALP: alkaline phosphatase; AMA: antimitochondrial antibody; ANA: antinuclear antibody; Anti-SMA: antismooth muscle antibody; AST: aspartate aminotransferase; BUN: blood urea nitrogen; INR: international normalized ratio.
